# Are Happiness and Life Satisfaction Different Across Religious Groups? Exploring Determinants of Happiness and Life Satisfaction

**DOI:** 10.1007/s10943-017-0481-2

**Published:** 2017-09-26

**Authors:** Kayonda Hubert Ngamaba, Debbie Soni

**Affiliations:** 1 Department of Social Policy and Social Work, University of York, Heslington, York, YO10 5DD UK; 2 School of Business, Education and Social Sciences, Messiah College, 1 College Ave, Mechanicsburg, 17055 PA USA

**Keywords:** Happiness, Life satisfaction, Religion, Religious differences, Culture

## Abstract

This study explores whether different religions experience different levels of happiness and life satisfaction and in case this is affected by country economic and cultural environment. Using World Value Survey (from 1981 to 2014), this study found that individual religiosity and country level of development play a significant role in shaping people’s subjective well-being (SWB). Protestants, Buddhists and Roman Catholic were happier and most satisfied with their lives compared to other religious groups. Orthodox has the lowest SWB. Health status, household’s financial satisfaction and freedom of choice are means by which religious groups and governments across the globe can improve the SWB of their citizens.

## Introduction

Maximizing citizens’ happiness and life satisfaction (i.e. subjective well-being) has been the preferred indicator of social progress (Greve 2010; Stiglitz et al. 2009; Veenhoven 2008), and researchers have suggested many factors that influence subjective well-being (SWB) including religion (Inglehart et al. 2008; Tovar-Murray 2011; Fleche et al. 2011). However, to date, the association between religion and SWB has appeared in a fragmented literature beset with methodological and conceptual difficulties. For example, most studies are limited to just looking at this issue in relation to Christianity and/or only look at one country. The aim of this study is to explore whether different religions experience different levels of happiness and life satisfaction and whether this is affected by country-specific/contextual factors such as cultural and economic environment. This study looks at a large number of different religious groups and across a vast range of countries using data from the World Value Survey. In this study, SWB is presented as a function of happiness and life satisfaction (Diener and Chan 2011; Kahneman and Deaton 2010). Happiness is most closely associated with emotions, feelings or moods (Gustafsson et al. 2009), and life satisfaction is concerned with people’s cognitive evaluations and judgements about their life, which might include evaluations of their work and/or personal relationships (Brickman and Campbell 1971; Coburn 2004; Diener et al. 1999).

A positive association between religion/spirituality and people’s SWB has been reported in empirical research. Most findings would tend to suggest that a religion/spirituality is of some benefit in terms of people’s sense of personal well-being and particularly so in areas such as: expressing emotions (Kim-Prieto and Diener 2009), encouraging good virtues (gratitude, caring and charitable actions) (McCullough et al. 2002), coping with adversity (Fischer et al. 2010), and social connections (Jung 2014) (see Table 1).

**Table 1:** Selected studies investigating the link between religion and subjective well-being (SWB)

Domains link to SWB	Authors and year	Topic investigated and findings	Targeted group
Expressing emotions			
	Kim-Prieto and Diener (2009)	Religion as a source of variation in the experience of positive and negative emotions: across countries, a study conducted amongst students from 49 nations studying in the USA, reported an association between religion and experience of emotions	Christian, Muslim, Hindu, Buddhist and Jewish; Cross-national: 49 nations
	McCullough et al. (2002), Metzl (2009)	Religion is associated with positive affect and well-being; it encourages the experience of certain emotions and discourages other emotions. Protestants Evangelical Christians seek to experience positive emotions at a high intensity compared to Christian Catholics	Christian Catholics, Protestants Evangelical Christians
	McCullough et al. (2002), Metzl (2009)	With their contemplative traditions, Buddhist may be encouraged to seek out emotions that are of low stimulation in their pleasantness	Buddhist
	Geschwind et al. (2011)	A randomized controlled trial links meditation to positive emotions	Buddhist
	Lutz et al. (2008)	Behavioural neuroscience studies on effects of meditation reported an association between greater religiosity and greater neural activation in the brain	Buddhist
	Sahraian et al. (2013)	Individuals with a more religious attitude experience more happiness	Muslims, Iran
	Kim-Prieto and Diener (2009)	Religion as a source of variation in the experience of positive and negative emotions: across countries, a study conducted amongst students from 49 nations studying in the USA, reported an association between religion and experience of emotions	Christian, Muslim, Hindu, Buddhist and Jewish; Cross-national: 49 nations
	Rozer and Kraaykamp (2013)	A higher level of SWB amongst Buddhists and Christians compared to Nonreligious people and people with Other religions	Christian, Muslim, Hindu, Buddhist and Jewish; Cross-national
	Ferriss (2002)	A greater percentage of Protestants who self-report as being “very happy” compared to Catholics or Jews	Protestants and Catholics
Encouraging good virtues: love, gratitude, caring and charitable actions			
	McCullough et al. (2002), Metzl (2009)	Christians, for example, encourage a certain attitude in response to the commandment “Love your neighbour”	Christians
	Ellison and Flannelly (2009), Tovar-Murray (2011)	Religious environment such as Christian centres can provide a discourse that discourages engagement in unhealthy behaviours	Christians
	McCullough et al. (2002)	Gratitude disposition has been found to be associated with positive affect and well- being, prosocial behaviours and traits, and religiousness/spirituality	Christian Catholics, Protestants Evangelical Christians
	Lyubomirsky and Layous (2013), Senf and Liau (2013)	Extraverted are happier, less depressed and more willing to express gratitude than neurotic	
	Tovar-Murray (2011)	A positive association between religious behaviours, spiritual beliefs, marital satisfaction, health and happiness amongst Jewish, Roman Catholics and Protestants in the USA	Jewish, Roman Catholics and Protestants, USA
Coping with adversity			
	Fischer et al. (2010)	Study reported a variation in well-being of Muslims and Christians due to the way these faith groups cope with adversity and stressful events. While Muslims were significantly more likely to seek social support from family, Christians were more likely to use intrapersonal coping strategies	Muslims and Christians
	Metzl 2009)	Religiosity increases resilience after a natural disaster (Hurricane Katrina)	Christians
	Chatters et al. (2011), Wells et al. (2012)	Religious belief might decrease the risk of stress, depression and suicidal thoughts	Christians
Social connections and attendance			
	Mochon et al. (2011)	While passionate believers benefit from their involvement, those with weaker beliefs are actually less happy than those who do not ascribe to any religion–atheists and agnostics	Christians, USA
	Ellison and Flannelly (2009)	A prospective nationwide study of African-American adults indicated that religious involvement is negatively associated with depression	Christians, USA
	Inglehart et al. (1992)	As institutions, religiosity may provide a support network	Christians
	Tewari et al. (2012)	Hindus’ participation in a long-duration mass gathering (such as a pilgrimage event) impacts well-being	Hindu, India
	Levin (2013)	Participation in synagogue activities was found to be significantly associated with less depression, better quality of life and more optimism	Jews, Israel
	Jung (2014)	Although the effect size is relatively small, religious attendance is associated with a higher level of happiness in South Korea. However, this positive effect holds only for women and only for Protestants	Protestants, Buddhists and Other Religions

Despite a large number of studies reporting a positive association between religion and SWB (see Table 1), questions have been raised about the representativeness of these findings because previous studies have been restricted to few religious groups and within-country analyses disregarding relevant contextual influences (Eichhorn 2013; Linley et al. 2009; Lobao and Hooks 2003; Lun and Bond 2013). Thus, several authors have called for: (1) a cross-national study of the link between religion and SWB and (2) inclusion into the analyses of national and social contexts (Lun and Bond 2013; Masud and Haron 2008).

Using a large number of different religious groups and across a large range of countries, this study explores whether different religions experience different levels of happiness and life satisfaction and whether this is affected by country-specific/contextual factors such as cultural and economic development. This study replicates the findings across countries using participants from a broad range of religious groups such as Buddhists, Hindus, Jews, Muslims, Christians, Other religious and Nonreligious groups. Moreover, this study investigates the role of variation within some religious groups such as Christian Roman Catholic, Protestant, Orthodox because these subgroups have different traditions and may have different intensity of emotions (Kim-Prieto and Diener 2009; McCullough et al. 2002). On top of affective components (i.e. happiness), this study investigates also the cognitive component (i.e. life satisfaction) in order to get a big and better picture of SWB across religions (Boldt 2006; Brockmann et al. 2009).

The list of major religions selected in this study was drawn from Pew Forum on Religion and comprised: Christians (31.4% of the world population), Muslims (23.2%), Hindus (15.0%), Buddhists (7.1%), Jews (0.2%), Other religious groups (0.8%, e.g. ancestral worshipping) and Nonreligious (16.4%, e.g. atheist, agnostic, people answering “none” or unaffiliated) (Pew_Research_Center 2015).

It is not easy to define each religious affiliation group, and this study does not intend to do so. Nevertheless, a Christian would be described as someone who believes in the person and ministry of Jesus Christ and who is a member of a Christian denomination. Amongst Christians, three big established groups were investigated: Roman Catholic, Orthodox and Protestants. Roman Catholic members recognize the Pope in the Vatican as the leader of the church and differentiate themselves from Orthodox and Protestants. The Orthodox, also known as Eastern Orthodoxy, identifies its roots in the early Church in Christian Era, and most adherents live in Russia, Eastern Europe and the Middle East. The Protestants are Christians who attempt to reform the Catholic Church in the early sixteenth century. Protestants included people who described themselves as Christian Protestants, Anglicans, Evangelical, Pentecostal, and so on. Muslims are those who believe in the teachings of Mohammad as a messenger of Allah; this group includes Shia and Sunni. A Buddhist supports the subscription to the Middle Way in accordance with what is outlined by Buddha in order to eventually achieve Enlightenment or Buddhahood as the goal. For the Hindu, however, adherence to the concepts of Hinduism, for example, is required in order for the devotee to achieve the all important Moksha and release from the Samsara cycle. Jews may describe themselves as people who trace their origins to the ancient Hebrew people of Israel and being part of a cultural community in which Judaism is the religion. While Hindus acknowledge multiple gods, Judaism, Christianity and Islam are in someway monotheistic religions (Pew_Research_Center 2015).

## Method

### Data Source

This study investigated the variability in happiness and life satisfaction across religious groups (Buddhists, Hindus, Jews, Muslims, Christians, Other Religious, and Nonreligious) using data from the World Value Survey (WVS). From 1981 to 2014, in collaboration with a European Values Study (EVS), the WVS carried out representative national surveys of more than 330,319 participants in 100 countries, using a common questionnaire to understand changing values and their impact on social and political life. In order to monitor these changes, the WVS executed six different surveys (1981–1984, 1989–1993, 1994–1999, 1999–2004, 2005–2007, 2010–2014) which in total, spanned approximately 33 years, that is from 1981 to 2014 (World-Values-Survey 2015). With an average of 1417 respondents, ranging from 240 to 3531 individuals, participants of each country were selected at random within the representative sample and interviewed face-to-face by a local field organization and supervised by WVS’s academic researchers (World-Values-Survey 2015). The ages ranged from 16 to 99 years with a mean of 42.28 years and standard deviation of 16.73. Pooled sample of all six waves of the WVS was verified, and a listwise deletion was applied to deal with missing data (Snijders and Bosker 2012); however, the complete cases represent a good percentage of more than 95%. For example, the happiness variable had some responses treated as missing data such as Don’t know (0.90%), No answer (0.27%), Not asked in survey (1.16%), Missing or Unknown (0.01%); the complete cases used for the happiness variable was 97.6%. Variables were scaled so that higher values reflected more of the positive characteristics. Nevertheless, because this study looked at a range of potential determinants of happiness and life satisfaction, the numbers of respondents were often lower due to missing data on some questionnaire items of interest.

Beside the main survey (i.e. World Value Survey); this study also used data taken from widely known sources that were combined with the main survey. Contextual influences are important in studies of religions across countries because religious people belong to countries where regional and national socio-economic and cultural factors apply. This study used GDP per capita drawn from the World Bank (World-Bank 2015), the Human Development Index (HDI) drawn from United Nations Development Programme (UNDP 2015), the Government Restrictions Index (GRI) and Social Hostilities Index (SHI) drawn from Pew Research Center (Pew_Research_Center 2015).

### Measures

#### Dependent Variables: Happiness and Life Satisfaction

This study used both common reliable SWB, namely happiness and life satisfaction. The combination of affective and cognitive components comes closest to people’s everyday experience and captures SWB better than one single item (Diener et al. 1999; Kahneman and Deaton 2010).

*Happiness* was assessed using a self-report scale 1–4 statement: taking all things together, would you say you are: On a scale of 1–4 if 1 = not at all happy; 2 = not very happy; 3 = quite happy; and 4 = very happy.

*Life satisfaction* was assessed using a self-report scale 1–10 question: “All things considered, how satisfied are you with your life as a whole these days?”, where “1” stands for “very dissatisfied” and “10” stands for “very satisfied”.

#### Independent Variables

*Religious affiliation group* Participants were asked to give the name of the religious denomination into which they belonged, and those who were not believers or affiliated to any religious groups selected Nonreligious. Dummy variable for each religious group was created (e.g. 1 = Muslim and 0 = otherwise). (See “Appendix 2” for the list of religious groups by country).

*Scale of incomes* 1 indicating the lowest income group, 2 the middle-income group, and 3 the highest income group in the country. “We would like to know in what group your household is, counting all wages, salaries, pensions and other incomes that comes in”. A dummy variable was created here and below for socio-economic factors (e.g. 1 = low-income scale and 0 = otherwise).

*Employment status* Full time, Part time, Self-employed, Retired, Housewife, a Student, Unemployed or part of some other employment category.

*Highest educational attainment level* Participants were asked to indicate their highest educational attainment level: from elementary, secondary to degree level.

*Socio-demographic factors* age group (i.e. 15–24, 25–34, 35–44, 45–54, 55–64, 65 and over), gender (i.e. men = 0, women = 1), marital status (i.e. married, living together, divorced, separated, widowed, single).

*Household’s financial satisfaction* was measured using the question: How satisfied are you with the financial situation of your household? (1 = completely dissatisfied and 10 = completely satisfied).

*Preference for income inequality* Respondents were asked to choose “1: if they wanted incomes to be made more equal” and “10: if they needed larger income differences as incentives”.

*State of health* All in all, how would you describe your state of health these days? If 1 = very poor, 2 = poor, 3 = fair, 4 = good and 5 = very good.

*Freedom of choice and control over life* How much freedom of choice and control do you feel you have over the way your life turns out? (1 = none at all and 10 = a great deal).

*Trust* “Generally speaking, would you say that most people can be trusted or that you need to be very careful in dealing with people?” The answer options were as follows: 0 = can’t be too careful or 1 = most people can be trusted.

*The importance of friends, family and leisure* indicates how important friends/leisure are in your life (1 = not at all important, 4 = very important).

*Attendance to religious services* “Apart from Weddings, Funerals and Christenings, how often do you attend religious services? 1 = never, 2 = once a year or less, 3 = on special holidays, 4 = once a month, 5 = every week”. A dummy variable was created (e.g. every week = 1 and 0 = otherwise).

*Importance of God* “How important is God in your life?” 1 = not important at all and 10 = very important. *Note* The question about the “importance of God” could be worded differently for certain groups that are not monotheistic, such as the Hindus.

*Religious person* (a person who manifests devotion to a deity): “Independently of whether you attend religious services or not, would you say you are: 1. A religious person, 2. Not a religious person, 3. An atheist”.

At country or aggregate level, this study controlled for GDP per capita, government restrictions to religions, social hostilities and geographical regions. For example, previous studies suggested that rich nations were happier than poor nations and that in the long run, the impact of growth was not significant (Easterlin 1974; Inglehart et al. 2008).

*GDP per capita* the sum of gross value added by all resident producers in the economy, plus any product taxed and lowered any subsidies not included in the value of the products (in current US dollars) (World-Bank 2015).

*Human Development Index (HDI)* drawn from the UNDP ranges from 0 to 1, with 0 indicating the lowest level of development and 1 the highest level of human development (UNDP 2015).

*The GRI* (government restrictions index), ranging from 0 to 10, with 10 indicating the highest level of government restrictions to religious practices or beliefs and 0 indicating the lowest level (Pew_Research_Center 2015).

*The SHI* (social hostilities index) also ranging from 0 to 10, with 10 indicating the highest level of social hostilities involving religion in a society and 0 indicating the lowest level (Pew_Research_Center 2015).

*Geographical regions* (1) Western Europe, (2) Eastern Europe and Former Soviet Union, (3) North America, (4) Latin America, (5) Asia, (6) sub-Saharan Africa, (7) Middle East and North Africa and (8) Australia. A dummy variable was created (e.g. Western Europe = 1 and 0 = otherwise) and tested the interaction between religious groups and different regions.

### Analysis

Using Stata 13.1 software (Stata 2013), this study explores the variability in happiness and life satisfaction across religious groups and whether the variability is affected by country-specific/contextual factors such as cultural and economic development. Nine religious groups were investigated: Buddhist, Hindu, Jew, Muslim, Roman Catholic, Orthodox, Protestant, Other religions and Nonreligious. Before to run the multilevel mixed-effects regression analysis, the one-way analysis of variance (ANOVA) was used to determine whether there are any significant differences between the means of these religious groups.

**Fig. 1:**
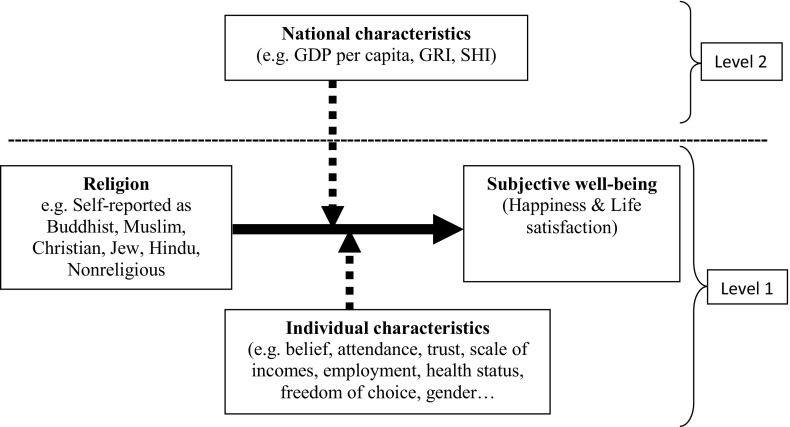
Visual representation of theoretical multilevel structure investigating the variability in happiness and life satisfaction across religions

A multilevel mixed-effects regression analysis (xtmixed Stata’s command) was used because WVS executed six different surveys from 1981 to 2014 (Snijders and Bosker 2012; Torres-Reyna 2014). The multilevel analysis methodology allows studying effects that vary by entity and estimates group level averages. This is important because the regular regression ignores the average variation between entities (Snijders and Bosker 2012). The mixed-effects analysis allows a wide variety of correlation patterns to be explicitly modelled. In this study, individuals who were affiliated to religious groups were nested by country (see Fig. 1) (Snijders and Bosker 2012).

The Models 1 and 2 were constructed for each dependent variable (i.e. happiness and life satisfaction). Correlations amongst variables were tested prior to analysis because highly correlated predictors might lead to multicollinearity and the last model of multivariate might be subject to suppressor effects or other statistical artefacts (Miller and Chapman 2001; Smith et al. 1992). There was no evidence of multicollinearity amongst the measured variables. Model 1 was the starting point where all religious groups were included without controlling for any independent variables. At this stage, the interaction between religion and geographical regions was tested. Model 2 extends Model 1 by controlling for covariates.

Variables used in this study were measured at different scales; thus, standardization procedures were applied to know which of the explanatory variables have a greater effect on happiness and life satisfaction. The thumb’s effect sizes (Cohen 1992) *r* ≤ .10 was used as a “small” effect size, *r* > .10 and ≤ .30 as a “medium” effect size, and *r* > .30 as a “large” effect size. The level of significance was: *p* < .001; *p* < .01; *p* < .05, and non-significant otherwise.

## Results

This study explored the variability in happiness and life satisfaction across main religious groups: Buddhist, Hindu, Jew, Muslim, Roman Catholic, Orthodox, Protestant, Other religions and Nonreligious. The average happiness (on a scale of 1 to 4) was slightly higher amongst Protestants (M = 3.21, SD = 0.72), followed by Buddhist (M = 3.17, SD = 0.63), Other religions (M = 3.17, SD = 0.72), Roman Catholic (M = 3.13, SD = 0.72), Jew (M = 3.06, SD = 0.73), Hindu (M = 3.05, SD = 0.78), Muslim (M = 3.03, SD = 0.76), Nonreligious (M = 3.02, SD = 0.71) and finally, Orthodox (M = 2.72, SD = 0.76) with the lowest happiness. There was a significant effect of religion on happiness at the *p* < .05 level for the different religious groups [F (8, 316630) = 1299.72, p = 0.001].

A similar pattern was seen for life satisfaction. The average life satisfaction (on a scale of 1 to 10) was slightly higher amongst Roman Catholics (M = 7.12, SD = 2.31), followed by Protestant (M = 7.07, SD = 2.33), Other religions (M = 6.97, SD = 2.26), Buddhist (M = 6.88, SD = 2.00), Jew (M = 6.85, SD = 2.23), Nonreligious (M = 6.62, SD = 2.30), Hindu (M = 6.23, SD = 2.50), Muslim (M = 6.16, SD = 2.55) and finally, Orthodox (M = 5.43, SD = 2.49) with the lowest life satisfaction. There was a significant effect of religion on life satisfaction at the *p* < .05 level for the different religious groups [F (8, 319261) = 2059.44, p = 0.001]. Amongst all religious groups, Orthodox had the lowest SWB. The correlations, tested prior to analysis, suggest a negative association between Orthodox and both happiness and life satisfaction (*r* = −0.144, *r* = −0.155, *p* < 0.01, respectively) (See “Appendix 1” for correlation between happiness, life satisfaction and other variables).

Table 2 presents the results of the multilevel mixed-effects regression analysis of happiness and life satisfaction. The results related to happiness are presented on the left, and those related to life satisfaction are presented on the right of Table 2.

**Table 2:** Multilevel mixed-effects regression analysis of happiness and life satisfaction across religious groups. *Source*: World-Values-Survey (2015)

	Happiness			Life satisfaction		
	Coef. (B)	Std. Err.	*p* value	Coef. (B)	Std. Err.	*p* value
Buddhist	0.001	0.003	0.651	−0.002	0.003	0.470
Hindu	0.002	0.003	0.521	0.009	0.003	0.004
Jew	−0.001	0.003	0.843	0.000	0.002	0.941
Muslim	0.013	0.006	0.046	−0.009	0.006	0.096
Roman Catholic	0.010	0.006	0.095	−0.001	0.005	0.925
Protestant	0.023	0.005	0.000	0.008	0.005	0.073
Orthodox	−0.001	0.005	0.776	−0.003	0.004	0.528
Other religious	0.006	0.003	0.031	0.004	0.002	0.083
Nonreligious	0.012	0.005	0.027	0.001	0.005	0.770
Full time	−0.017	0.007	0.019	0.002	0.006	0.786
Part time	−0.006	0.004	0.181	−0.001	0.004	0.766
Self-employed	−0.013	0.005	0.007	−0.003	0.004	0.483
Retired	0.000	0.005	0.946	0.002	0.005	0.718
Housewife	0.010	0.006	0.085	0.014	0.005	0.005
Students	0.005	0.004	0.277	0.011	0.004	0.005
Unemployed	−0.031	0.005	0.000	−0.018	0.004	0.000
Other employment	0.002	0.003	0.404	0.001	0.002	0.635
Elementary education	−0.010	0.004	0.005	0.004	0.003	0.252
Secondary education	−0.010	0.004	0.015	0.003	0.004	0.466
University education	−0.010	0.004	0.004	0.005	0.003	0.100
Gender (female)	0.023	0.002	0.000	0.020	0.002	0.000
Married	0.079	0.022	0.000	0.028	0.019	0.144
Together	0.023	0.011	0.030	0.010	0.009	0.317
Divorced	−0.013	0.008	0.094	−0.010	0.007	0.185
Separated	−0.013	0.006	0.031	−0.011	0.005	0.040
Widowed	−0.018	0.011	0.087	−0.008	0.009	0.375
Single	−0.005	0.019	0.773	−0.017	0.017	0.327
Age 16–24	0.041	0.018	0.025	0.006	0.016	0.703
Age 25–34	0.015	0.020	0.452	−0.012	0.018	0.504
Age 35–44	0.001	0.019	0.946	−0.021	0.017	0.220
Age 45–54	−0.002	0.017	0.892	−0.017	0.015	0.276
Age 55–64	0.004	0.015	0.784	−0.005	0.014	0.722
Age 65–over	0.017	0.014	0.242	0.001	0.013	0.923
Low-income scale	−0.026	0.004	0.000	−0.017	0.003	0.000
Middle-income scale	0.001	0.003	0.707	0.006	0.003	0.044
High-income scale	0.006	0.003	0.071	0.015	0.003	0.000
Financial satisfaction	0.175	0.002	0.000	0.385	0.002	0.000
Inequality preferences	0.003	0.002	0.094	0.011	0.002	0.000
State of health	0.262	0.002	0.000	0.141	0.002	0.000
Freedom of choice	0.091	0.002	0.000	0.197	0.002	0.000
Meaning of life	0.005	0.002	0.007	−0.012	0.002	0.000
National pride	0.082	0.002	0.000	0.047	0.002	0.000
Trust	0.021	0.002	0.000	0.017	0.002	0.000
Friends important	0.036	0.002	0.000	0.013	0.002	0.000
Family important	0.048	0.002	0.000	0.024	0.002	0.000
Leisure important	0.041	0.002	0.000	0.013	0.002	0.000
Weekly Rel. attend	0.020	0.006	0.001	0.016	0.005	0.002
Monthly attend	0.002	0.004	0.671	0.003	0.004	0.428
Special days attend	−0.001	0.005	0.835	0.007	0.004	0.092
Yearly attend	−0.003	0.005	0.457	0.005	0.004	0.186
Never attend	0.002	0.005	0.726	0.015	0.004	0.001
Importance of God	0.013	0.003	0.000	0.040	0.002	0.000
Religious person	−0.021	0.002	0.000	−0.008	0.002	0.000
GDP	−0.052	0.007	0.000	−0.052	0.006	0.000
Gini coefficient	−0.052	0.006	0.000	−0.045	0.005	0.000
HDI	−0.090	0.018	0.000	0.024	0.014	0.096
GRI	0.054	0.012	0.000	0.046	0.010	0.000
SHI	0.007	0.008	0.393	−0.019	0.007	0.007
Western Europe	0.215	0.241	0.372	0.134	0.150	0.373
Eastern Europe	−0.213	0.248	0.390	−0.177	0.155	0.251
North America	0.281	0.302	0.353	0.079	0.188	0.674
Latin America	0.131	0.260	0.614	0.161	0.162	0.322
Asia	−0.007	0.261	0.979	−0.069	0.162	0.669
Africa	−0.313	0.262	0.233	−0.344	0.165	0.036
Middle east	−0.383	0.264	0.147	−0.251	0.165	0.127
Australia	0.206	0.302	0.496	0.058	0.188	0.758
Intercept	−0.170	0.253	0.502	0.158	0.157	0.316
N	237,443					

Standardized variables; significant *p* < .001, .01, .05

In terms of happiness, the multilevel analysis showed a positive association with being protestant, female, married, younger (16 to 24 years old), household’s financial satisfaction, state of health, freedom of choice, national pride, trust, importance of friends, family and leisure, weekly religious attendance and importance of God. On the other hand, being unemployed and in low-income scale groups were negatively associated with happiness.

With regard to life satisfaction, a similar trend has been observed. The multilevel analysis showed a positive association with being female, household’s financial satisfaction, state of health, freedom of choice, national pride, trust, importance of friends, family and leisure, weekly religious attendance and importance of God. On the other hand, being unemployed, in low-income scale groups and meaning of life were negatively associated with life satisfaction.

Nevertheless, according to Cohen’s rules of thumb (Cohen 1992) only three factors were above the “small” effect size (> 0.10). State of health, household’s financial satisfaction and freedom of choice showed “medium” effect sizes and were positively associated with happiness and life satisfaction.

## Discussion

This study explores whether different religions experience different levels of happiness and life satisfaction and in case this is affected by country-specific/contextual factors such as economic and cultural environment.

In terms of happiness, individuals who described themselves as Protestants and Buddhists were characterized by high experiences of happiness compared to any other groups. With regard to life satisfaction, Roman Catholics, Protestants and Buddhists were more satisfied with their lives than any other groups. On the other hand, those who described themselves as Orthodox were less happy and less satisfied with their lives compared to any other group. Variability in happiness and life satisfaction across religious groups has been supported empirically, despite the fact that some religious groups have never been investigated across countries. For example, our results reported higher levels of happiness and life satisfaction amongst Protestants compared to other religious groups, as some cross-national studies have stated (Ferriss 2002; Rozer and Kraaykamp 2013). This study found differences in happiness between Protestants and Roman Catholics. Emotional well-being seems to be more prominent amongst Protestants rather than Roman Catholics. In line with previous studies, Christian Protestants seek to experience positive emotions at a high intensity compared to Christian Catholics (Ferriss 2002; Metzl 2009). Nevertheless, with regard to life satisfaction, both Protestants and Roman Catholics were equally satisfied with their lives. Our results found that Protestants were not the only people to be characterized by higher levels of happiness and life satisfaction, but these levels could be found in women, who have higher religious attendances amongst Protestants. These findings may explain why a positive association between attendance to religious services and happiness has been found in women and Protestants in South Korea but not amongst Buddhists, Catholics and other religious groups (Jung 2014).

Our results demonstrated that within the Christian faith, people who described themselves as Orthodox were characterized by lower levels of happiness and life satisfaction compared to Nonreligious and any other religious groups. An important question has been asked in the literature, can people’s religiosity make them really happier or are they happier because they belong to a happy nation or their happiness through religiosity can mainly be derived through conforming to the standard in their country (Eichhorn 2013; Linley et al. 2009; Lobao and Hooks 2003; Lun and Bond 2013)? Our results provide empirical support suggesting that religiosity and country level of development both play an important role in shaping people’s happiness and life satisfaction. For example, religious members living in developed regions such as Western Europe, North America and Australia were happier and more satisfied with their lives than those living in less developed regions such as Eastern Europe, Africa and the Middle East. Interestingly, people who describe themselves as Orthodox were less happy and less satisfied with their lives and were mainly located in Eastern Europe and Former Soviet Union. Nevertheless, with the same GDP per capita, people living in Latin America are happier and more satisfied with their lives than people living in Eastern Europe. Living in Latin America, a region traditionally Roman Catholic and Protestant might explain the high levels of happiness and life satisfaction compared to Eastern Europe where the collapse of communism has left a spiritual vacuum (Inglehart et al. 2008). Without a doubt, this argument is challenged with surveys of China and Vietnam, suggesting that despite the remaining presence of communist parties, those countries enjoy high economic growth and might show, in the long run, an increase in SWB than Eastern Europe (Knight and Gunatilaka 2010). While further research needs to investigate the underlying cause of low levels of happiness and life satisfaction amongst Orthodox, our study found that Orthodox living in Eastern Europe self-reported lower levels of happiness and life satisfaction compared to Orthodox living in Latin America. In line with previous studies, this may suggest that there are differences in the experience of happiness and life satisfaction across different religious groups (Kim-Prieto and Diener 2009). On the other hand, country level of development plays an important role in shaping people’s SWB (Howell and Howell 2008).

The most significant factors driving happiness and life satisfaction include state of health, household’s financial satisfaction, income ranking position, unemployment, freedom of choice, national pride, trust, importance of friends, family, leisure, being a female and weekly religious attendance (see Table 2). Nevertheless, when the Cohen’s rules of thumb (Cohen 1992; Wright 1992) was applied, most factors seem to have “small” effect size (*r* ≤ 0.10). Thus, the most significant factors driving happiness and life satisfaction were state of health, household’s financial satisfaction and freedom of choice.

Health status is positively associated with higher happiness and life satisfaction. In line with previous studies, good health is associated with greater well-being, while setbacks in health have negative effects on happiness and life satisfaction. For example, people who have painful chronic conditions and those who become seriously disabled have permanently lower levels of SWB compared to their counterparts who are not disabled (Headey 2010). Our multilevel analysis showed a positive association between health status and both happiness and life satisfaction even after controlling for several factors including GDP per capita, relative income, psychological factors, socio-economic and demographic factors (Miret et al. 2014; Fleche et al. 2011). Thus, improving people’s health status is one means by which governments across the globe can improve the subjective well-being (SWB) of their citizens.

This study found that the magnitude of the association between household’s financial satisfaction and SWB was medium, positive and significantly stronger amongst different religious groups. The results on household’s financial satisfaction support the “need theory” as a universal approach across religions and suggest that income not only allows individuals to purchase goods and services (Howell and Howell 2008), but it also goes hand in hand with happiness and life satisfaction (Ng and Diener 2014). Absolute and mostly relative income plays an important role in influencing happiness and life satisfaction (Boyce et al. 2010; Easterlin 1974, 2005). If GDP per capita can no longer be used as the best indicator of people’s living standard (Stiglitz et al. 2009), being in a country where basic needs (e.g. health, education and income indispensable for a decent standard of living) are provided plays an important role in shaping people’s SWB (Inglehart et al. 2008).

Emancipative values such as freedom of choice, gender equality and tolerance have been associated with life satisfaction (Inglehart et al. 2008). Everybody shall have the right to freedom of choice including freedom to have, to adopt a religion or to express feelings and emotions. Religious groups that promote good values such as freedom of choice, freedom of emotions, gratitude, and social connections may improve the SWB of their members (Fischer et al. 2010; Jung 2014; Kim-Prieto and Diener 2009).

It is important to recognize four limitations of this research. First, all variables used in this study were measured by single items. Although researchers have used the same single-item happiness (Inglehart et al. 2008; Lun and Bond 2013), it is important to replicate the current findings with better-validated multi-item scales (Fisher et al. 2016). Second, this study examined as much as possible explanatory variables including socio-cultural and demographic factors, but there might be other important factors that were not measured in this study. Third, this research reported that people from some religious groups, such as Orthodox, were less happy and less satisfied with their lives, further studies are encouraged to investigate the underlining causes. Also, further work must be done to expand the research of subgroups of certain of these religious groups such as: Sunnis and Shia Muslims, Messianic Jews.

In conclusion, by investigating the variability in happiness and life satisfaction across a large number of religious groups, this study has provided empirical support suggesting that religiosity and country level of development both play a significant role in shaping the SWB of people. Religious groups that promote good values such as freedom of choice, freedom of emotions, gratitude and social connections may improve the SWB of their members. Health status, household’s financial satisfaction and freedom of choice are means by which governments across the globe can improve the subjective well-being of their citizens.

## Appendix 1

See Table 3.

**Table 3:** Correlation between happiness and life satisfaction and other factors. *Source*: World-Values-Survey (2015)

	Happiness	Life satisfaction
Happiness	1.0000	
Life satisfaction	0.4704	1.0000
Buddhist	0.0281	0.0198
Hindu	−0.0016 ns	−0.0266
Jew	0.0011 ns	0.0083
Muslim	−0.0171	−0.0932
Roman Catholic	0.0535	0.1192
Protestant	0.0790	0.0593
Orthodox	−0.1444	−0.1555
Other religious	0.0233	0.0233
Nonreligious	−0.0203	0.0024 ns
Full time	0.0248	0.0477
Part time	0.0179	0.0209
Self-employed	0.0197	−0.0043
Retired	−0.0641	−0.0248
Housewife	0.0205	0.0143
Students	0.0453	0.0345
Unemployed	−0.0584	−0.0874
Other employment	−0.0041	−0.0187
Elementary education	−0.0404	−0.0491
Secondary education	0.0237	−0.0039
University education	0.0644	0.0770
Gender (female)	0.0077	0.0089
Married	0.0485	0.0106
Together	0.0364	0.0458
Divorced	−0.0557	−0.0371
Separated	−0.0336	−0.0168
Widowed	−0.0978	−0.0591
Single	0.0114	0.0049
Age 16–24	0.0494	0.0317
Age 25–34	0.0274	−0.007 ns
Age 35–44	0.0033	−0.0118
Age 45−54	−0.0259	−0.0181
Age 55–64	−0.0337	−0.0054
Age 65–over	−0.0366	0.0020 ns
Low-income scale	−0.1427	−0.1796
Middle-income scale	0.0506	0.0483
High-income scale	0.1187	0.1532
Financial satisfaction	0.3413	0.5606
Inequality preferences	0.0483	0.0551
State of health	0.3727	0.3011
Freedom of choice	0.2465	0.3992
Meaning of life	0.0378	−0.0018 ns
National pride	0.1633	0.1260
Trust	0.0588	0.0754
Friends important	0.1229	0.0866
Family important	0.1118	0.0650
Leisure important	0.1407	0.1280
Weekly Rel. attend	0.0795	0.0292
Monthly attend	0.0073	0.0177
Special days attend	−0.0378	−0.0395
Yearly attend	−0.0245	−0.0131
Never attend	−0.0383	−0.0123
Importance of God	0.0610	0.0320
Religious person	−0.0365	−0.0002 ns
GDP	0.1318	0.1823
Gini coefficient	0.0715	0.0434
HDI	0.0621	0.1774
GRI	−0.0790	−0.1146
SHI	−0.0585	−0.0997
Western Europe	0.0569	0.1168
Eastern Europe	−0.1975	−0.1963
North America	0.0706	0.0820
Latin America	0.0902	0.1879
Asia	0.0384	0.0066
Africa	0.0208	−0.0888
Middle east	−0.0558	−0.0867
Australia	0.0572	0.0675

Pairwise correlations, significant *p* < .01; *ns* non-significant

## Appendix 2

See Table 4.

**Table 4:** List of religious groups by country. *Source*: World-Values-Survey 2015

	Buddhist	Hindu	Jew	Muslim	Rom Cath	Protestant	Orthodox	Other relig	Nonrelig	Total
Albania	6	13	90	706	650	184	204		141	1994
Algeria	0	0	0	2476	0		0		0	2476
Andorra	0	9	0	12	545	10	3	10	412	1001
Azerbaijan	0	0	5	2787	2	7	55		135	2991
Argentina	74	11	72	5	4826	130	28	244	979	6369
Australia	65	26	42	31	1216	1825	80	33	1531	4849
Bangladesh	10	302	1	2684	17	2	1		4	3021
Armenia	1	0	3	1	14	13	2658	14	331	3035
Bosnia	0	0	3	485	154	1	248	1	293	1185
Brazil	10	0	3	3	2934	791	109	137	602	4589
Bulgaria	2	3	2	224	14	10	1296		497	2048
Belarus	0	0	4	6	316	32	2272	1	921	3552
Canada	21	9	14	39	1676	853	30	262	1115	4019
Chile	1	6	8	0	3613	525	140	120	1231	5644
China	314	1	0	75	30	177	0	22	5508	6127
Taiwan	809	45	217	1	41	473	252	597	785	3220
Colombia	2	0	2	2	8223	800	127	169	1219	10,544
Croatia	0	1	5	14	989	4	14		147	1174
Cyprus	0	0	3	931	9	3	982	8	108	2044
Czech Rep.	0	0	1	0	797	75	0		1120	1993
Dominican Rep.	0	0	0	0	245	55	0	11	98	409
Ecuador	0	0	0	0	753	162	0	4	282	1201
El Salvador	28	0	0	0	406	288	0		200	922
Ethiopia	1	0	6	158	23	291	971	24	8	1482
Estonia	6	0	0	6	33	214	525	19	1706	2509
Finland	0	0	85	63	325	2111	30	6	371	2991
France	5	0	2	49	411	26	2	2	496	993
Georgia	1	2	76	55	1708	48	1403	54	147	3494
Palestine	0	0	0	997	2		0	1	0	1000
Germany	5	2	2	87	1249	1852	35	33	2774	6039
Ghana	1	1	1	417	531	1723	201	100	72	3047
Guatemala	1	0	0	2	560	308	0	33	90	994
Hong Kong	273	3	0	2	67	259	0	47	1592	2243
Hungary	0	0	13	6	1791	687	17	12	463	2989
India	172	7845	34	957	169	185	49	276	288	9975
Indonesia	0	0	1	2785	65	136	0	13	7	3007
Iran	0	0	0	5081	0	32	4	32	42	5191
Iraq	0	0	0	6159	16	23	9		0	6207
Israel	0	0	1023	114	0	39	0	5	0	1181
Italy	2	1	0	0	885		0	2	121	1011
Japan	2587	2	3	0	36	77	97	188	3346	6336
Kazakhstan	2	2	1	767	14	9	399		304	1498
Jordan	0	0	0	3510	29	61	20	1	0	3621
South Korea	1679	3	5	8	1065	1460	25	168	2557	6970
Kyrgyzstan	3	2	30	2111	9	19	170	3	189	2536
Lebanon	0	0	0	622	261	13	133	100	0	1129
Latvia	1	0	3	4	222	233	217		447	1127
Libya	0	0	0	2058	0		0	35	0	2093
Lithuania	2	1	1	1	778	20	42		132	977
Malaysia	461	193	3	1509	84	150	0	17	37	2454
Mali	1	8	11	1426	27	8	1	16	5	1503
Mexico	8	3	13	5	7935	843	39	139	1743	10,728
Moldova	0	0	16	2	41	49	2662	12	172	2954
Morocco	0	3	7	3634	2	1	1	1	0	3649
Netherlands	7	7	3	54	595	137	57	147	1607	2614
New Zealand	15	20	8	12	410	1613	3	80	736	2897
Nigeria	1	4	26	2076	1082	2875	216	148	284	6712
Norway	6	0	1	17	25	1590	10	20	431	2100
Pakistan	0	3	0	3096	0	1	0		601	3701
Peru	3	6	5	1	4184	669	0	44	448	5360
Philippines	0	0	0	126	2711	262	0	8	254	3361
Poland	1	0	1	0	2911	27	31	12	104	3087
Puerto Rico	25	0	10	0	1071	315	0	147	297	1865
Romania	3	0	8	9	250	234	3912	2	26	4444
Russia	22	2	12	367	18	58	4180	127	3587	8373
Rwanda	5	1	3	305	1639	753	32	95	201	3034
Saudi Arabia	0	5	0	1457	0	28	0	6	3	1499
Singapore	791	320	6	898	230	330	0	325	544	3444
Slovakia	0	0	0	0	1126	149	3		242	1520
Viet Nam	383	1	3	1	151	26	1	1156	769	2491
Slovenia	3	1	0	41	2085	49	58	10	820	3067
South Africa	32	595	98	658	1960	8943	113	880	2128	15,407
Zimbabwe	1	1	0	20	475	1694	15	51	243	2500
Spain	10	2	3	7	5042	37	9	54	1091	6255
Sweden	6	7	27	38	70	3873	10	54	1064	5149
Switzerland	1	1	10	24	1793	1417	7	102	359	3714
Thailand	2639	1	2	65	7	2	0	6	7	2729
Trinidad and Tobago	5	435	0	122	404	850	8	28	126	1978
Tunisia	0	0	0	1205	0		0		0	1205
Turkey	0	0	6	7669	14	20	3	18	519	8249
Uganda	0	1	0	170	366	442	4	7	11	1001
Ukraine	7	4	17	18	328	51	3275		1382	5082
Macedonia	0	0	4	505	10	5	1084	2	422	2032
Egypt	0	0	0	5686	0	363	0	1	0	6050
Great Britain	5	8	2	40	106	326	4	26	496	1013
Tanzania	0	1	42	469	330	219	58	23	20	1162
USA	42	13	156	26	2097	3221	25	1104	1665	8349
Burkina Faso	0	1	2	818	473	120	3	84	16	1517
Uruguay	3	1	6	0	997	202	0	150	1615	2974
Uzbekistan	1	3	1	1426	1	4	45		9	1490
Venezuela	2	1	0	0	1777	155	2	15	414	2366
Yemen	0	0	0	1000	0		0		0	1000
Serbia and Montenegro	0	0	7	33	48	9	1063		45	1205
Zambia	2	4	1	20	513	694	2	182	82	1500
Serbia	1	0	2	125	153	15	1788	29	300	2413
Montenegro	0	0	0	273	80	2	884	1	43	1283
Bosnia	0	0	2	317	157	1	4		312	793
Total										324,320
